# Modular Orthopaedic Tissue Engineering With Implantable Microcarriers and Canine Adipose-Derived Mesenchymal Stromal Cells

**DOI:** 10.3389/fbioe.2020.00816

**Published:** 2020-07-22

**Authors:** Chara Simitzi, Maja Vlahovic, Alex Georgiou, Zalike Keskin-Erdogan, Joanna Miller, Richard M. Day

**Affiliations:** ^1^Centre for Precision Healthcare, Applied Biomedical Engineering Group, UCL Division of Medicine, University College London, London, United Kingdom; ^2^Department of Biomolecular and Sports Sciences, Coventry University, Coventry, United Kingdom; ^3^Cell Therapy Sciences Ltd., University of Warwick Science Park, Coventry, United Kingdom; ^4^Eastman Dental Institute, University College London, London, United Kingdom

**Keywords:** mesenchymal stromal cells, modular tissue engineering, cell microcarriers, osteogenic differentiation, chondrogenic differentiation

## Abstract

Mesenchymal stromal cells (MSC) hold significant potential for tissue engineering applications. Modular tissue engineering involves the use of cellularized “building blocks” that can be assembled via a bottom-up approach into larger tissue-like constructs. This approach emulates more closely the complexity associated hierarchical tissues compared with conventional top-down tissue engineering strategies. The current study describes the combination of biodegradable porous poly(DL-lactide-co-glycolide) (PLGA) TIPS microcarriers with canine adipose-derived MSC (cAdMSC) for use as implantable conformable building blocks in modular tissue engineering applications. Optimal conditions were identified for the attachment and proliferation of cAdMSC on the surface of the microcarriers. Culture of the cellularized microcarriers for 21 days in transwell insert plates under conditions used to induce either chondrogenic or osteogenic differentiation resulted in self-assembly of solid 3D tissue constructs. The tissue constructs exhibited phenotypic characteristics indicative of successful osteogenic or chondrogenic differentiation, as well as viscoelastic mechanical properties. This strategy paves the way to create *in situ* tissue engineered constructs via modular tissue engineering for therapeutic applications.

## Introduction

The ability of mesenchymal stromal cells (MSC) from various adult sources to self-renew and differentiate into multiple tissue types of mesoderm, including bone, adipose and cartilage, together with their hypo-immunogenicity, has attracted attention for potential use in the repair of tissues (Caplan, 1991; Dimarino et al., 2013). This covers immune-mediated and degenerative disease, including bone and cartilage damage (Lazarus et al., 1995; Horwitz et al., 1999), tissue fibrosis (Lim et al., 2017), cardiovascular and peripheral artery disease, including limb ischemia (Liew and Brien, 2012; Qadura et al., 2018; Yong et al., 2018), and neurodegenerative disease (Joyce et al., 2010; LoFurno et al., 2018). Furthermore, the intrinsic secretory activity of MSC results in a plethora of immunomodulatory, anti-apoptotic and pro-angiogenic factors that contribute to a pro-regenerative environment (Caplan, 2007).

The implementation of MSC in tissue engineering (TE) strategies to replace damaged tissue has been widely associated with the use of scaffold components that provide a framework to guide tissue regeneration, whilst ensuring controlled retention of transplanted cells at the target site. Scaffold-based TE approaches originally involved pre-shaping biomaterial components into desired shapes followed by cell loading. Such top-down TE approaches result in non-homogenous cell distribution throughout the scaffold that do not recapitulate the complex hierarchical environment of tissues (Sittinger et al., 2004). To address this, the use of additive manufacturing, consisting of 3D (bio)printing to deliver precise quantities of cells and materials into pre-defined configurations mimicking complex tissues and organs has been shown to be technically feasible using a variety of biomaterials and cells (bioink) (Murphy and Atala, 2014). To overcome the challenge of maintaining the structural integrity of printed tissue during implantation, 3D (bio)printing *in situ* directly at the site of tissue defects has been proposed (Hong et al., 2018; Ashammakhi et al., 2019). However, this approach is mostly limited to sites that are easily accessible (e.g., open wounds), making it less compatible with minimally invasive surgery.

Conversely, modular TE strategies address these limitations by using cellularized building blocks that can assemble “bottom-up” into larger tissue-like constructs. This approach allows introduction of tuneable microarchitecture and complexity [reviewed in Guillotin and Guillemot (2011)]. The driving force of assembly relates to either surface properties of the biomaterial units and/or the intrinsic ability of cells to aggregate. Patterning the individual units provides predefined hierarchical organization that can facilitate maturation of the TE construct toward functionally relevant tissue architectures (Guillotin and Guillemot, 2011). Examples of modular TE include assembly of cell-laden hydrogel units (McGuigan and Sefton, 2006; Du et al., 2008; Fernandez and Khademhosseini, 2010), cell sheets (Elloumi-Hannachi et al., 2010), cell encapsulation units (Correia et al., 2020), and the 3D printing of cell aggregates (Norotte et al., 2009; Skardal et al., 2010). Other approaches exploit assembly of specific cells to develop micro-tissue units derived from bio-fused cells and micro-objects/biomaterials (Urciuolo et al., 2013; Leferink et al., 2019). The modular approach offers particular value in the case of complex tissues, such as epithelized cartilage (Guillotin and Guillemot, 2011; Leferink et al., 2014; Dikina et al., 2018). The strategy of using cellularized scaffolding elements could be especially helpful in conditions where the construct needs to be configured to fill irregular-shaped defects, such as in the case of bone fillers, intra-osseous injections or joint defects in orthopaedics to facilitate regeneration. Existing approaches for these types of application include injectable natural polymer-based hydrogels [reviewed in Liu et al. (2017)], with cell types including osteoblasts, chondrocytes, and MSC (Kassem et al., 1997). However, mechanically soft materials, such as injectable hydrogels synthesized from natural biomaterials, typically have limited biomechanical properties (Liu et al., 2017). The potential benefit of using self-assembling units for injectable systems as fillers for bone tissue engineering applications has been recently described (Leferink et al., 2014).

Spherical microparticulate scaffolds are particularly well suited for modular TE strategies that require minimally invasive delivery into poorly accessible sites. Their geometry has previously been shown to be particularly well suited for use as cell-microcarriers, a feature that has been previously explored in tissue engineering using PLGA microcarriers and MSC (Bouffi et al., 2010; Morille et al., 2013, 2016; Penna et al., 2013). Furthermore, the use of PLGA microparticles as components in modular assembly of tissue engineered constructs has been previously reported (Jaklenec et al., 2008; Fleischer et al., 2017). However, each of these approaches have involved pre-functionalization of the polymer scaffold component to become biologically active. For products intended for clinical translation, the inclusion of biologically active ingredients into the scaffold device component increases complexity relating to formulation development and manufacturing process optimization. The presence of a biologically active component may also increase potential risk to patient safety, which will require more extensive analytical characterization and non-clinical assessment due to the product following a different regulatory pathway compared with TE approaches that incorporate non-functionalized scaffold device components.

The current study describes the use of non-functionalized biodegradable porous PLGA microcarriers for modular TE using canine adipose-derived MSC (cAdMSC). The PLGA microcarriers were prepared using the thermally induced phase separation (TIPS). Surface porosity, which is well controlled via the fabrication process, facilitates MSC adhesion without the need of additional protein coating. Following optimization of cell attachment efficiency, the cellularized PLGA microcarriers were cultured in transwell insert plates to create a hierarchical environment for 21 days under culture conditions that induce either chondrogenic or osteogenic differentiation. After 21 days, cellularized PLGA TIPS microcarriers in both differentiation conditions (but not in control medium) self-assembled into solid 3D composite tissue constructs with phenotypic characteristics indicative of osteogenic or chondrogenic differentiation. The mechanical properties of the 3D tissue constructs exhibited viscoelastic characteristics. By using cellularized PLGA TIPS microcarriers as self-assembly units the study demonstrates for the first time the *ex vivo* formation of osteogenic and chondrogenic neo-tissue like composite based on cAdMSC and non-functionalized PLGA microcarriers via a modular approach.

## Materials and Methods

### Fabrication of PLGA TIPS Microcarriers Using Thermally Induced Phase Separation

Poly(DL-lactide-co-glycolide) TIPS microcarriers were prepared using thermally induced phase separation as previously described (Blaker et al., 2008). Briefly, 10% w/v PLGA (Purasorb PDLG7507; Corbion) was dissolved in dimethyl carbonate (DMC; Sigma-Aldrich) using magnetic stirring overnight. The polymer solution was fed into a Nisco Var D encapsulator unit (Nisco Engineering, Switzerland) fitted with a stainless steel sapphire-tipped nozzle with a 100 μm orifice. The flow rate was controlled by a syringe pump (Pump 11; Harvard Apparatus) set at a constant rate of 2 mL/min. The vibration frequency of the nozzle was kept at 2.70 kHz and the amplitude of frequency at 100%. Liquid polymer droplets were ejected into a 1 L polypropylene beaker (Azlon Plastics) containing liquid nitrogen. The frozen droplets were allowed to equilibrate in the liquid nitrogen. Samples were lyophilized using an Edwards MicroModulyo freeze dryer (Thermo Fisher Scientific) for 24 h to allow sublimation of residual solvent. For experiments conducted in the current study, PLGA TIPS microcarriers were sieved to 250–425 μm diameter (Endecotts^TM^ Stainless Steel Test Sieve, Fisher Scientific, Loughborough, United Kingdom) and aliquoted into glass vials (50 ± 2 mg).

### Characterization of PLGA Microcarriers

For ultrastructural characterization of the PLGA TIPS microcarriers, samples were mounted on aluminum stubs using adhesive carbon tabs and sputter coated with gold (Polaron E5000). Samples were viewed using a Hitachi S3400N scanning electron microscope (SEM) at 5 keV. An image processing algorithm (ImageJ, National Institutes of Health, Bethesda, MD, United States) was used to determine the size and shape of the PLGA TIPS microcarriers by evaluating diameter and shape descriptors, including aspect ratio and roundness from top view SEM images using the traced circumference tool in ImageJ.

The number of PLGA TIPS microcarriers per unit mass was quantified from the mean value of six samples from static image analysis using the Morphologi G3 system (Malvern Panalytical Ltd., United Kingdom).

### Isolation of cAdMSC From Adipose Tissues

Canine adipose-derived MSC were isolated from falciform and inguinal adipose tissue from six canine donors with different background breeds (Table 1). All samples were collected as part of procedures being performed for preparation of cells for unrelated therapeutic clinical applications. Excess cells from the unrelated therapeutic preparations were collected with the informed consent of the owners.

**TABLE 1:** Canine donor demographics: breed, sex, age and source of fat used for isolating stromal cells used in this study.

Donor #	Breed	Sex	Age	Fat source
1	Cocker Spaniel	M	7	Falciform
2	Border Collie	M	11	Falciform
3	Labrador	M	13	Inguinal
4	Airedale Terrier	M	1	Falciform
5	Border Collie	F	7	Falciform
6	Labrador	F	9	Falciform

The adipose tissues were visually examined and any signs of abnormalities, such as blood clots, skin or hairs were removed and the cells isolated using a collagenase digestion method (Krešić et al., 2017). The tissue samples were washed three times, with phosphate buffered saline (PBS) and mechanically minced with surgical scissors before digestion with collagenase I (Sigma Aldrich C0374) and agitation with a magnetic stirrer at 37°C for 30 min. The digested adipose tissue was filtered through a 100 μm Steriflip filtration tube (Merck) to remove undigested tissue and large debris. The samples were centrifuged to produce a stromal vascular fraction (SVF). The SVF pellet contained a heterogenous population of cells such as MSC, leukocytes and erythrocytes. Erythrocytes were lysed with ammonium chloride potassium (ACK) buffer (Thermo Fisher Scientific) by resuspending the SVF in 5 ml of ACK and incubating for 5 min at room temperature. After incubation, the ACK was diluted with 45 ml PBS. The ACK/SVF was centrifuged at 1500 × *g* for 5 min. The supernatant was removed, the erythrocyte-free pellet was re-suspended in proliferation medium and plated into a tissue culture treated flask (Falcon). cAdMSC were incubated at 37°C and 5% CO_2_ with medium changes every 2 days and passaged until a homogeneous cell line was established (passage 3). All experiments were conducted with cells between passage 3–5.

### Verification of cAdMSC Differentiation Toward Osteogenic and Chondrogenic Lineages

For osteogenic differentiation, cAdMSC were seeded at 5 × 10^4^ cells/ml and cultured in low glucose media (LGFCS) (10% FBS, 1% antibiotic and antimycotic, Dulbecco’s Eagle Medium-low glucose) (Thermo Fisher Scientific) until cells reached 80% confluency. After 3 days, the culture medium was replaced with StemPro^®^ Osteogenesis Differentiation medium (Thermo Fisher Scientific) for 14 days, with the medium replenished every 3 days. Osteogenic differentiation was evaluated by staining with Alizarin Red S (Acros Organics, United States). Quantification of the staining was performed by dye extraction using an aqueous solution of 20% (v/v) methanol (Acros Organics) and 10% (v/v) acetic acid (Acros Organics). The absorbance was measured at 450 nm wavelength.

For chondrogenic differentiation, cAdMSC were seeded at 2 × 10^5^ cells/100 μl in U-bottom 96 well plates that were non-tissue culture treated (low attachment) to enable high density culture conditions, as described elsewhere for chondrogenic differentiation and incubated for 48 h to form spheroids (Zhang et al., 2010; PromoCellGmbH, 2015). After the spheroids were formed, the LGFCS was removed and the spheroids washed two times with PBS before adding 100 μl of StemPro Chondrogenesis Differentiation Kit (Thermo Fisher Scientific). Control spheroids received 100 μl LGFCS. The medium was changed every 3 days. After 21 days the medium was removed, the cells rinsed with PBS and fixed with 4% formaldehyde solution for 30 min. To stain for the presence of synthesized proteoglycans indicative of chondrogenic differentiation, the spheroids were incubated in Alcian blue staining solution prepared in 0.1 N HCL for 45 min at room temperature, rinsed with 0.1 N hydrochloric acid and washed 3× in diH_2_O. The spheroids were placed under a glass coverslip and imaged using light microscopy.

### Attachment of cAdMSC to PLGA TIPS Microcarriers

Poly(DL-lactide-co-glycolide) TIPS microcarriers were pre-wetted as previously described to assist with cell attachment (Wright et al., 2015). The wetting solution consisted of 10% (v/v) absolute ethanol in proliferation medium [Dulbecco’s Modified Eagles Medium (DMEM)-high glucose (4.5 g/L) with GlutaMAX^TM^ (Gibco, United Kingdom), 10% v/v fetal bovine serum (FBS; Life Sciences, Seradigm, United States) and 1% antibiotic and antimycotic (0.25 μg/ml Amphotericin B, 100 units/ml penicillin and 100 μg/ml streptomycin) (Gibco)]. 50 mg PLGA TIPS microcarriers in a 7 ml polystyrene container were incubated in 5 ml of the wetting solution under rotation at 37°C for 18 h. Wetting of the PLGA TIPS microcarriers was confirmed by their sedimentation in the container prior to washing twice with fresh proliferation medium.

Attachment of cAdMSC to PLGA TIPS microcarriers was investigated using three cell concentrations (0.5 × 10^6^, 1 × 10^6^, 2 × 10^6^ cells/ml) under three different conditions: (i) static, (ii) semi-dynamic conditions on a plate shaker for 18 h with 30 s shaking every hour, or (iii) semi-dynamic conditions on a plate shaker for 18 h with 30 s shaking every 15 min. Cells were co-cultured with 50 mg PLGA TIPS microcarriers (approximately 9000 microcarriers) in low-attachment 6-well plates (Corning, United Kingdom). After 18 h incubation, the microcarriers were gently rinsed with PBS to remove non-attached cells and the quantity of cells attached to the microcarriers determined using a NucleoCounter^®^ NC-200^TM^ automated cell counter (ChemoMetec A/S, Denmark), as recommended by the manufacturer.

To evaluate the quantity of cells attached per individual PLGA TIPS microcarrier after 6 h, the cellularized microcarriers were fixed with 4% formaldehyde in PBS, permeabilized with 0.5% Triton X-100 in PBS and the nuclei stained with Hoechst (Thermo Fisher Scientific, United Kingdom). Cells were imaged using an inverted fluorescence microscope (Leica DM16000B) and the number of nuclei per microcarrier quantified using ImageJ (“Cell Counter” plugin). The quantity of cells was expressed as cells per unit of microcarrier surface area (cells/cm^2^) by dividing the number of cells counted by the surface area of a known quantity of microcarriers. The results represent the mean value of six experiments (*n* = 60–70 PLGA microcarriers per condition).

### Viability of cAdMSC Attached to PLGA TIPS Microcarriers

The viability of cells attached to the microcarriers after 24 h of culture was determined by incubating the cellularized microcarriers with 10% (v/v) Presto Blue Cell Viability Reagent (Invitrogen, United Kingdom) in 2 ml proliferation medium in a 6-well plate. After incubating for 2 h at 37°C, 100 μl of the supernatant was transferred into black-walled 96-well plates (Corning, United Kingdom) and the fluorescence intensity was measured at 560 nm (exc) and 620 nm (em).

Live-Dead staining (Thermo Fisher Scientific, United Kingdom) was used to qualitatively assess cell viability on the PLGA TIPS microcarriers after 21 days incubation. Samples were imaged immediately using an inverted fluorescence microscope (Leica DM16000B).

### Differentiation of cAdMSC on PLGA TIPS Microcarriers

Simulation of *in situ* confinement and differentiation of cellularized PLGA TIPS microcarriers post-delivery in a tissue cavity was performed using transwell insert plates (96-well MultiScreen, Millipore, Thermo Fisher Scientific, United Kingdom. cAdMSC (2 × 10^6^) were seeded onto 50 mg PLGA TIPS microcarriers in a 6-well low attachment plate under semi-dynamic conditions (30 s shaking every 15 min) for 18 h. The cellularized microcarriers were transferred into the 96-well transwell insert plate and incubated in proliferation medium for 24 h at 37°C and 5% CO_2_. The medium was replaced with either 400 μl osteogenic differentiation medium (STEMPRO osteogenic differentiation kit, Thermo Fisher Scientific, United Kingdom), chondrogenic differentiation medium (ChondroMAX Differentiation Medium, Sigma) or proliferation (control) medium. The osteogenic and chondrogenic media were changed every 3–4 and 2–3 days, respectively.

Differentiation of cAdMSC to an osteogenic-like phenotype was evaluated at days 14 and 21 using Alizarin Red S staining (Acros Organics). Samples were washed with PBS, fixed with 4% formaldehyde for 30 min at room temperature and washed with diH_2_O. Alizarin stain was added for 3 min and washed with diH_2_O. The samples were imaged using a stereomicroscope (Leica MZ10F). Quantification of the staining was performed by dye extraction using an aqueous solution of 20% (v/v) methanol (Acros Organics) and 10% (v/v) acetic acid (Acros Organics). The absorbance of the extracted dye was measured at 450 nm wavelength.

Differentiation of cAdMSC to a chondrogenic-like phenotype was evaluated at Day 21 by Alcian blue staining. The samples were rinsed with PBS and fixed with 4% formaldehyde for 30 min. After fixation, the samples were rinsed with PBS and stained with Alcian blue. The samples were rinsed with 0.1 N HCl and subsequently with diH_2_O. The samples were imaged using a stereomicroscope.

Cell and collagen deposition around the PLGA TIPS microcarriers was evaluated by histology. After 21 days in differentiation medium, the cellularized PLGA TIPS microcarriers were washed with PBS and fixed with 4% formaldehyde for 2 h at 4°C. The samples were washed with PBS and dehydrated by immersion in serially graded ethanol solutions (75–100% v/v), followed by incubation in Histo-clear II (National Diagnostics, United States). The specimens were subsequently embedded in Paraplast-Plus (Sigma-Aldrich). 6 μm thick sections were cut and de-waxed, then stained with hematoxylin and eosin or with Picro-Sirius Red Solution (Abcam, United Kingdom). Samples were imaged using a NanoZoomer (Hamamatsu Photonics, Japan) or inverted photomicroscope (IX81; Olympus, United Kingdom), respectively.

Cell interaction with the PLGA TIPS microcarriers was assessed by SEM. After 21 days in differentiation medium the cellularized PLGA TIPS microcarriers were washed with PBS and fixed with 4% formaldehyde for 2 h at 4°C. The samples were washed with PBS and dehydrated by immersion in serially graded ethanol solutions (20–100% v/v) for 5 min each. The samples were immersed in hexamethyldisilazane (Sigma Aldrich) for 2 min and sputter coated with gold (Polaron E5000). Samples were viewed using a Philips XL30 field emission SEM.

### Dynamic Mechanical Analysis

Mechanical testing of the cAdMSC-PLGA TIPS microcarrier constructs was evaluated at Day 21 using a Discovery DMA 850 (TA Instruments, New Castle, DE, United States). Samples measuring 4–5 mm in diameter and 1 mm height were analyzed. The aspect ratio of the sample was kept consistent for all sample measurements to ensure a similar loading mode. Compression testing was conducted at 37°C using cyclic sinusoidal load mode, which varied from 0.05 to 40 Hz frequencies at 5 Hz intervals. Groups were pre-loaded to 0.001 N force and dynamically tested under small deformation (0.1% strain) compression to ensure that the data collected was repeatable.

### Statistical Analysis

Experimental data were tested for Gaussian distribution and subjected to one-way or two-way ANOVA followed by Tukey’s test for multiple comparisons between pairs of means, using GraphPad Prism (version 7.0). Statistically significant differences between experimental group was indicated by ^∗^*p* < 0.05, ^∗∗^*p* < 0.01, ^∗∗∗^*p* < 0.001, and ^****^p < 0.0001. The results are expressed as mean ± standard error of the mean (SEM).

## Results

### Characterization of PLGA TIPS Microcarriers

**FIGURE 1:**
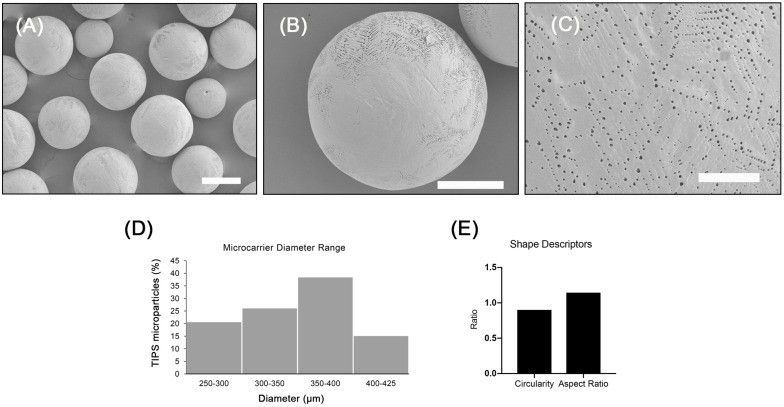
Porous PLGA TIPS microcarriers fabricated using thermally induced phase separation. **(A–C)** SEM images of the surface of PLGA TIPS microcarriers (scale bar in A, B and C is 250, 150, and 25 μm, respectively). **(D)** Frequency of different sized PLGA TIPS microcarriers present in the samples investigated. **(E)** Quantitative evaluation of circularity and aspect ratio of PLGA TIPS microcarriers investigated.

The microcarriers investigated exhibited a highly porous surface topography (Figures 1A–C). Higher magnification images of the microcarriers revealed many of the pores were distributed in a chevron pattern (Figure 1C). The size of the surface pores ranged from 0.17 to 2.40 μm, which corresponds with previous studies using the same composition of TIPS microparticles (Simitzi et al., 2020). The porous structure of the microcarriers results from thermally induced separation that occurred during the manufacturing process (Blaker et al., 2008). Size distribution of the microcarriers ranged from 300 to 400 μm (Figure 1D). Circularity and aspect ratio values were close to 1 (1.14 ± 0.20 and 0.90 ± 0.12, respectively), indicating that PLGA TIPS microcarriers were highly spherical (Figure 1E). Pre-wetting the PLGA TIPS microcarriers prior to cell attachment reduced their mean diameter to 254 ± 82 μm.

### cAdMSC Isolation and Differentiation

**FIGURE 2:**
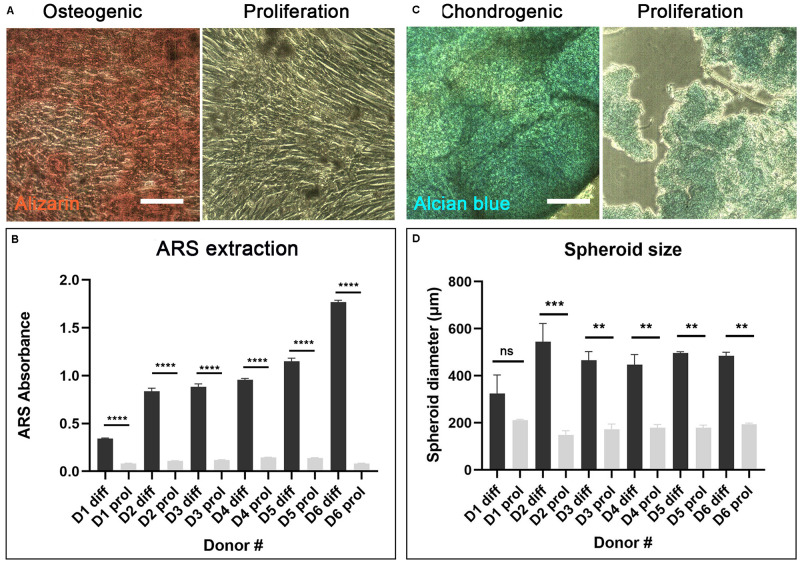
Differentiation of cAdMSC toward osteogenic or chondrogenic lineages. **(A)** Light microscopy of cAdMSC incubated for 14 days in osteogenic differentiation (diff) or proliferation medium (prol) and stained for Alizarin red (scale bar 100 μm). **(B)** Quantification of Alizarin red stain (ARS) extracted from cAdMSC at day 14; the level of Alizarin red staining for all donors was significantly higher (*p* < 0.0001) in cAdMSC cultured in osteogenic differentiation medium compared with cells cultured in proliferation medium. **(C)** Light microscopy of cAdMSC incubated for 21 days in chondrogenic differentiation (diff) or proliferation (prol) medium and stained for Alcian blue (blue) (scale bar 100 μm). **(D)** Spheroid diameter of cAdMSC from six donors (D1–D6) incubated in chondrogenic (diff) or proliferation (prol) medium for 21 days; spheroid diameter was significantly higher for donor 2 (*p* < 0.001) and for donors 3–6 (*p* < 0.01) in cAdMSC cultured in chondrogenic differentiation medium compared with cells cultured in proliferation medium. **p < 0.01, ***p < 0.001, and ****p < 0.0001.

After 7–10 days culture, cAdMSC from all donors grew as confluent monolayers. The cells exhibited a polarized to polygonal fibroblast-like shape. This morphology was maintained during further sub-passaging. cAdMSC from all six donors differentiated toward osteogenic-like lineage after 14 days in osteogenic differentiation medium, as indicated by significantly increased positive staining for Alizarin red compared with cells cultured for the same length of time in proliferation medium (Figure 2A and Supplementary Figure S1A). The level of Alizarin staining was measured in cAdMSC derived from each donor (Figure 2B). The level of Alizarin red staining was significantly increased in cAdMSC cultured in osteogenic differentiation medium compared with cells cultured in proliferation medium (*p* < 0.0001).

Canine adipose-derived MSC from all six donors differentiated toward chondrogenic-like lineage after 21 days in chondrogenic differentiation medium. The cells from all donors formed large spheroids of similar size that stained positive for Alcian blue (Figure 2C and Supplementary Figure S1B). Control cAdMSC maintained in proliferation medium under the same conditions formed significantly smaller (*p* < 0.01 or *p* < 0.001 for all donors except for donor 1) and more fragile spheroids that exhibited weak staining for Alcian blue (Figure 2D).

### cAdMSC Attachment to PLGA TIPS Microcarriers

**FIGURE 3:**
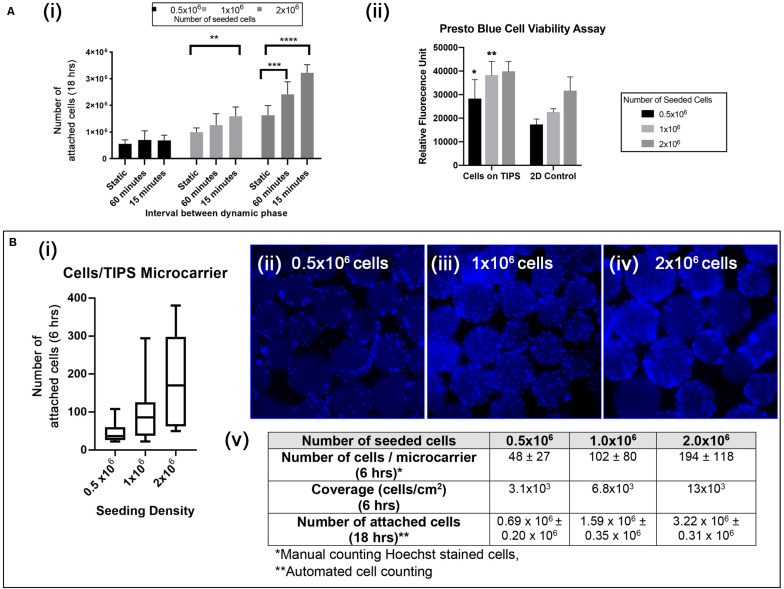
**(Ai)** Quantification of cAdMSC attachment to the surface of PLGA TIPS microcarriers after incubation for 18 h under different experimental conditions. Data shown represent the mean value for cells derived from six donors (**p < 0.01, ***p < 0.001, and ****p < 0.0001). **(Aii)** Metabolic activity of cAdMSC incubated on PLGA TIPS microcarriers for 24 h assessed using the Presto Blue assay (statistical comparison was performed between the cells-on-TIPS and 2D condition for each concentration; *p < 0.05, **p < 0.01). **(Bi)** Quantification of cell attachment per microcarrier after 6 h incubation using three cell seeding concentrations (*n* = 60–70 TIPS microcarriers counted per group). **(Bii–iv)** Fluorescence microscopy of Hoechst stained cAdMSC attached to the surface of the microcarriers after 6 h incubation. **(Bv)** Summary of the cell attachment results at different time points expressed as total number of cells per 50 mg PLGA TIPS microcarriers or number of cells/per microcarrier.

Optimal conditions for attachment of cAdMSC to PLGA TIPS microcarriers were investigated by varying the concentration of cells (0.5 × 10^6^, 1 × 10^6^, 2 × 10^6^) and culture conditions (static or intermittent 30 s plate shaking at 15 min and 60-minute intervals). Under static conditions not all of the cells from all donors attached to the microcarriers after 18 h incubation (Figure 3Ai and Supplementary Figure S2A). Intermittent shaking of the plates increased the proportion of cells attached to the microcarriers after 18 h incubation (Figure 3Ai and Supplementary Figure S2B). The best conditions tested for cell attachment consisted of adding 2 × 10^6^ cells and incubating with 30 s plate shaking at 15-minute intervals (Figure 3Ai and Supplementary Figure S2C). (Supporting data in Supplementary Figure S2 show the cell attachment for all six donors).

The level of cell metabolic activity (measured by Presto Blue staining) in samples containing cells attached to microcarriers was higher compared with cells cultured on TC plastic (significantly higher for 0.5 × 10^5^ and 1 × 10^5^ cells/ml, Figure 3Aii). This result is likely to reflect the higher surface area available with microcarrier groups compared with the TC plastic groups resulting in a great number of cells when confluence was reached. Staining of the cell nuclei confirmed that increasing the seeding concentration of cells corresponded with more cells attaching to the microcarriers (Figures 3Bii–iv). However, at higher seeding concentrations the cells became less evenly distributed, as shown by the larger interquartile range of cells per microcarrier (Figures 3Bi,v).

### Formation of Osteogenic-Like Tissue Engineered Constructs

The long-term behavior of cAdMSC on PLGA TIPS microcarriers and their capacity to differentiate into an osteogenic-like lineage was evaluated *in vitro*. Cellularized microcarriers were cultured in transwell inserts that confined the microcarriers into a multi-layered 3D porous configuration. The pore size of the transwell insert mesh (60 μm) allowed non-attached cells and debris to dissociate from the 3D construct.

**FIGURE 4:**
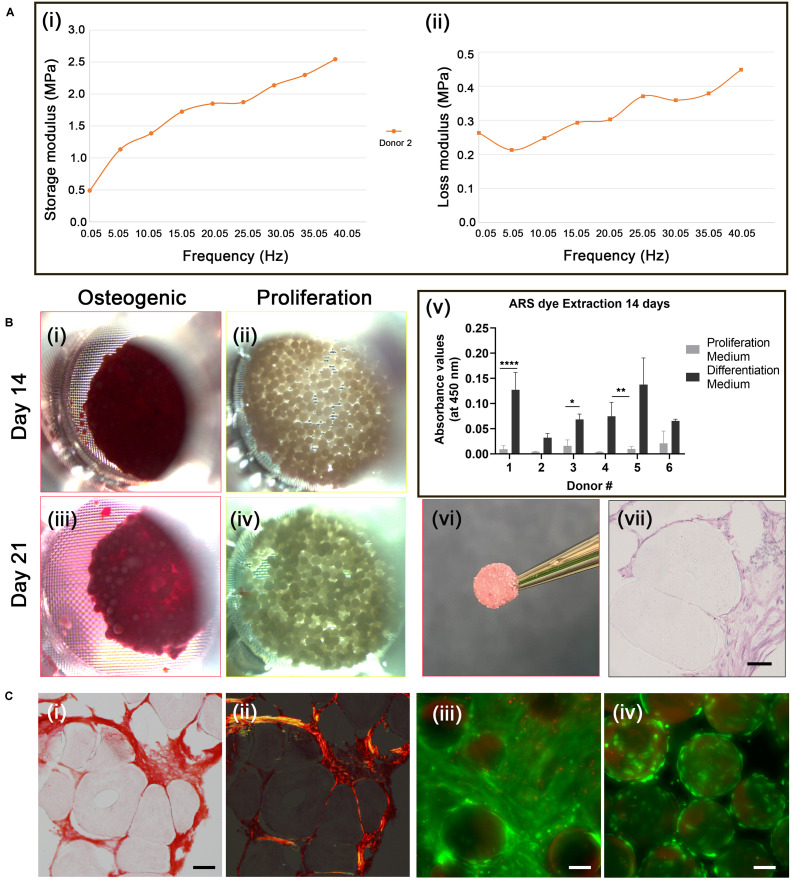
Osteogenic differentiation of cAdMSC on PLGA TIPS microcarriers. **(A)** Dynamic Mechanical Analysis. Storage modulus **(i)** and loss modulus **(ii)** of the disc structure consisting of cellularized PLGA TIPS microcarriers derived from one donor sample after 21 days incubation in osteogenic differentiation medium, measured by dynamic mechanical analysis in compression mode. **(B)** Alizarin red staining. Light microscopy images of cellularized PLGA TIPS microcarriers after incubation in osteogenic differentiation medium **(i,iii)** or proliferation medium **(ii,iv)**; **(v)** Quantification of Alizarin red staining via dye extraction after 14 days; the level of Alizarin red staining for donors 1,3 and 4 was significantly higher (*p < 0.05, **p < 0.01, and ****p < 0.0001) in cAdMSC cultured in osteogenic differentiation medium compared with cells cultured in proliferation medium; **(vi)** The disc-like structure consisting of cellularized PLGA TIPS microcarriers after 21 days incubation in osteogenic differentiation medium; **(vii)** Histology of the sectioned disc-like structure showing cellular infiltration between microcarriers throughout the structure (hematoxylin and eosin staining; scale bar 50 μm). **(C) (i)** Picrosirius red staining of collagen in the disc structure consisting of cellularized PLGA TIPS microcarriers after 21 days incubation in osteogenic differentiation medium) and **(ii)** polarized light microscopy of the sample (red: collagen fibers, green: collagen type III, yellow: collagen type I; scale bar 50 μm). Live/Dead staining of cellularized PLGA TIPS microcarriers after 21 days in culture in differentiation medium **(iii)** or in proliferation medium **(iv)** showing cells remained viable in both culture conditions and formed bridges between the cellularized microcarriers in the differentiation conditions (scale bar 100 μm).

Incubation of the cellularized microcarriers in differentiation medium resulted in the formation of clusters that fused into solid disc-like composite structures composed of the cells and microcarriers. Control samples incubated in proliferation medium resulted in limited clustering only at the highest cell seeding concentration, with noticeably less extensive fusion occurring compared with the samples incubated under osteogenic differentiation conditions. At day 14 large discs occupying most of the bottom surface of the transwell insert were observed with cAdMSC derived from five out of the six donors. However, at day 21 samples from all donors had fused into the disc-like structures. The 3D discs consisted of densely packed cellularized PLGA TIPS microcarriers. The volume of the disc occupying the transwell insert became reduced at day 21 compared with day 14, indicating active remodeling of the TE construct and degradation of the microcarriers over time. All of the discs became biconcave and strong enough to be handled with forceps without fragmenting. The control group incubated in proliferation medium resulted in only a few clusters of cellularized microcarriers at day 21, with none evident at day 14. Mechanical analysis of one donor sample from the differentiated culture group using dynamic mechanical analysis (DMA) in compression mode revealed that the discs exhibited viscoelastic characteristics, exhibiting both an elastic (storage modulus, E’) and a viscous behavior (loss modulus, E”) (Figure 4A). The elastic modulus was higher than the loss modulus at all frequencies studied.

Cell differentiation in the disc-like structures was assessed on day 14 and 21 using Alizarin red staining. AdMSC stained positively for Alizarin red at both time-points (Figures 4Bi,iii). No positive staining for Alizarin was present in the samples incubated in proliferation medium (Figures 4Bii,iv). The level of extracted Alizarin red staining in cAdMSC cultured on PLGA TIPS microcarriers was measured at day 14 (Figure 4Bv). Cells from all donors showed a trend toward increased Alizarin staining when maintained in differentiation medium compared with cells maintained in proliferation medium.

Histology of the discs at day 21 showed collagen-rich matrix deposition around the PLGA TIPS microcarriers (Figures 4Ci,ii). Collagen was present in the form of both small thin fibers (collagen type III; stained green) and thicker fibers (collagen type I; stained yellow). Deformation of the PLGA TIPS microcarriers was evident (Figures 4Bvii, 4Ci), which corresponded with the remodeling of the cellularized PLGA TIPS microcarriers observed at the macroscopic level.

Live/Dead staining of the samples at day 21 showed that the majority of cells maintained in the proliferation and differentiation medium were viable (Figures 4Ciii,iv). Samples incubated in the differentiation medium resulted in multiple layers of cells that formed a covering of the microcarriers (Figure 4Ci).

### Formation of Chondrogenic-Like Tissue Engineered Constructs

Chondrogenic differentiation of cAdMSC on PLGA TIPS microcarriers was evaluated using a similar experimental approach to that used for osteogenic differentiation. Cells from the six donors were cultured on PLGA TIPS microcarriers in either differentiation medium or proliferation medium for 21 days.

**FIGURE 5:**
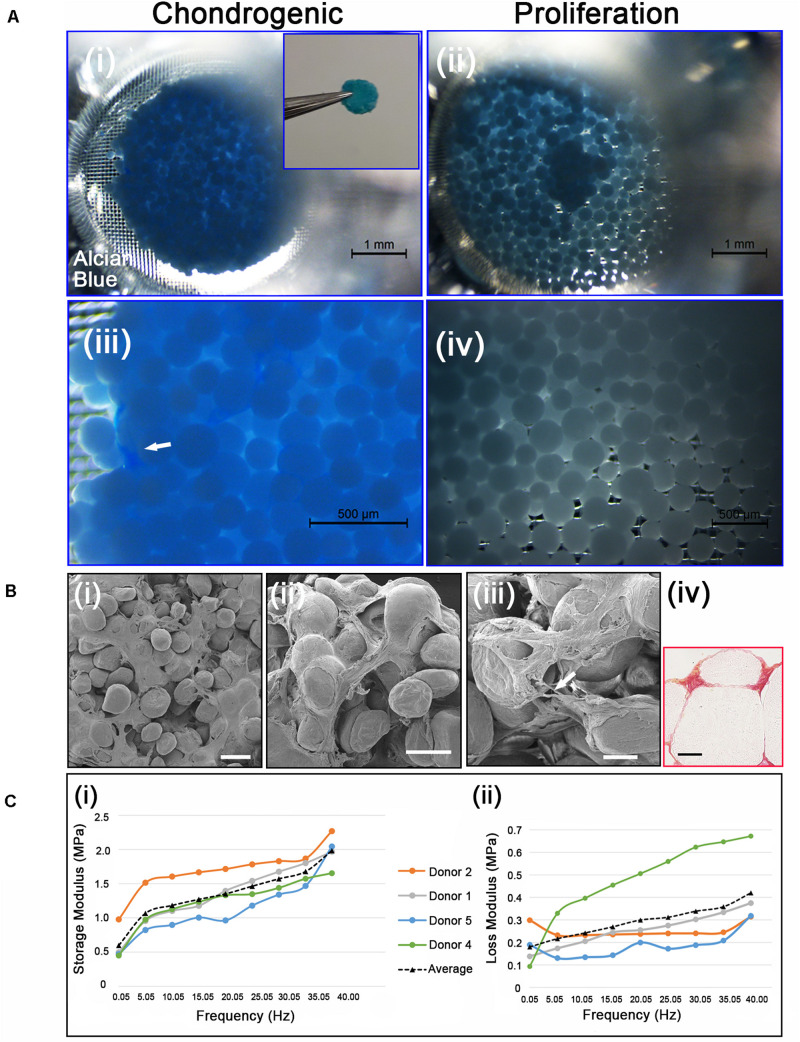
**(Ai,iii)** Chondrogenic differentiation of cAdMSC on PLGA TIPS microcarriers incubated for 21 days in chondrogenic differentiation medium or **(ii,iv)** proliferation medium and stained with Alcian blue (Inset shows the disc structure consisting of cellularized PLGA TIPS microcarriers after 21 days incubation in chondrogenic differentiation medium). **(Bi–iii)** SEM of the disc structure consisting of cellularized PLGA TIPS microcarriers after 21 days incubation in chondrogenic differentiation medium (scale bar 100 μm). Arrow in panel **(B) (iii)** shows a cell bridging two PLGA TIPS microcarriers; **(iv)** Picrosirius red staining of collagen in the disc-like structure consisting of cellularized PLGA TIPS microcarriers after 21 days incubation in chondrogenic differentiation medium (red: collagen fibers, yellow: cytoplasm; scale bar 50 μm). **(C) (i)** Storage modulus and **(ii)** loss modulus of the disc-like structure consisting of cellularized PLGA TIPS microcarriers after 21 days incubation in chondrogenic differentiation medium measured by dynamic mechanical analysis in compression mode.

Samples incubated in differentiation medium displayed extensive clustering, forming a solid disc consisting of cells and microcarriers, similar to that observed with the osteogenic differentiation medium. The quantity of cellularized PLGA TIPS microcarriers used covered the bottom surface area of the transwell insert at the time of cell seeding. When the cellularized PLGA TIPS microcarriers were maintained in chondrogenic differentiation medium they fused together with increasing time. At day 21, the disc-like structure of cellularized PLGA TIPS microcarriers had remodeled and was reduced in diameter compared with the cellularized PLGA TIPS microcarriers incubated in proliferation medium for the same period, which did not show similar evidence of remodeling or reduction in diameter (Figure 5Ai vs. 5Aii). Inter-donor variation in disc-like structure formed was observed, with discs forming in 5 out of the 6 donors. The discs from the donors 3, 4, 5 and 6 had a diameter of 4.3 ± 0.4 mm. The donor cells that did not form a disc resulted in clusters of cellularized PLGA TIPS microcarriers. Qualitative observations revealed the disc-like structures from four donors were sufficiently robust to be handled with forceps and required considerable manual force to be broken.

Cell differentiation was assessed on day 21 using Alcian blue staining, which stains the proteoglycan components of the extracellular matrix associated with chondrocytes. The disc-like structures of cellularized PLGA TIPS microcarriers maintained in the chondrogenic medium stained positively for Alcian blue (Figures 5Ai, 5Aiii). Within the discs there were dense regions of positively stained GAGs (arrow in Figure 5Aiii). In contrast, samples incubated in proliferation medium formed small clusters that exhibited either weak or no staining (Figures 5Aii, 5Aiv). Ultrastructural analysis of the cellularized microcarriers at day 21 in differentiation medium showed that cells covered the PLGA TIPS microcarriers and extended between adjacent microcarriers within the structure (Figures 5Bi–iii). Histology showed collagen-rich matrix deposition around the PLGA TIPS microcarriers (Figure 5Biv). Dynamic mechanical analysis of the discs of cellularized PLGA TIPS microcarriers from four donors robust enough to be handled after 21 days in the chondrogenic differentiation medium exhibited characteristics of a viscoelastic material, showing both an elastic (storage modulus, E’) and a viscous behavior (loss modulus, E”; Figure 5C). Both storage and loss moduli were frequency-dependent and for all frequencies tested, storage modulus was greater than loss modulus. The mean storage modulus ranged from 0.45 to 2.30 MPa, while the mean loss modulus ranged from 0.10 to 0.67 MPa.

## Discussion

The current study investigated whether biodegradable TIPS microcarriers are feasible for use in modular orthopaedic tissue engineering applications. Spherical microcarriers were fabricated from PLGA polymer using thermally induced phase separation technique. This method is highly versatile and can be tailored to produce desired characteristics for size, stiffness, porosity and surface texture (Foong et al., 2010). The study used cells derived from canine donors collected as part of procedures being performed for preparation of cells for unrelated therapeutic clinical applications, demonstrating the technical feasibility of collecting and using clinically isolated samples for the cellular component of the cellularized “building blocks.” This observation, combined with the TIPS microparticles being readily available for clinical use, provides an opportunity for future studies to investigate the efficacy of the proposed therapeutic system *in vivo* in a comparative clinical science setting. Adopting such an approach will also allow the impact of donor-to-donor variability on efficacy to be explored in a real-life treatment setting, which will generate new knowledge for human and veterinary medicine.

Following pre-wetting, the PLGA TIPS microcarriers used in the current study were biocompatible for cAdMSC, making it unnecessary to add ECM protein coating to enhance cell attachment. The established pre-wetting process involves hydrophilization of the hydrophobic polymer with a solution containing ethanol (Wright et al., 2015). The presence of ethanol in the wetting solution, a weak solvent for PLGA that does not dissolve the microcarriers during the wetting process, resulted in shrinkage of the hydrophilized microcarriers. Similar macroscopic shrinkage of PLGA scaffolds resulting from exposure to ethanol has been reported previously for TIPS microcarriers and other PLGA scaffolds (Gualandi, 2011; Simitzi et al., 2020). Co-culture of the microcarriers with cAdMSC under optimized conditions resulted in efficient cell attachment within 18 h. The study screened two major parameters affecting cell attachment, namely the seeding concentration of cells and static-dynamic culture conditions. The process used to enable cell interaction with the microcarriers was reliant on sedimentation of cells onto the surface of the microcarriers. To increase the likelihood of an evenly distributed quantity of cells attaching to all the microcarriers in the vessel required the microcarriers to be dispersed in a single layer to maximize the surface area exposed. Too many microcarriers would result in them stacking and reduce the likelihood of those below the surface layer interacting with the cells, whereas too few microcarriers would result in cells falling into gaps between the microcarriers and not attaching. Static-dynamic culture consisting of brief shaking of the plate followed by 15-minute static incubation was found to increase the number of cells attaching to the microcarriers’ surface. This is likely to have resulted from the shaking phase increasing the likelihood of cells interacting with the surface of the microcarriers. The interval between the dynamic phase of the incubation period when the plate was re-shaken to lift the microcarriers and re-disperse the unattached cell suspension was also important. The more frequent plate shaking with 15-minute intervals was associated more cell attachment compared with the plates shaken at 60-minute intervals. Sufficient time was also required for the cells to interact with the surface of microcarrier and adhere before shaking, which would otherwise dislodge non-attached cells. As expected, the seeding concentration of cells also influenced the number of cells attaching to the microcarriers. At lower seeding concentrations, shaking had a lower impact on the number of cells attaching and proliferating. Seeding 1 × 10^6^ cells in a dynamic mode produced the same number of attached cells as seeding 2 × 10^6^ cells under static conditions. The optimal conditions for achieving the maximum number of attached cells was achieved when 2 × 10^6^ cells were seeded and shaken every 15 min; therefore, those conditions were used for the differentiation experiments. For all cell attachment conditions investigated, the number of cells increased by 1.6–1.8-fold between 6 and 24 h, indicating the substrate and culture conditions for the attachment process were also conducive for cell proliferation.

Following cell attachment, the cellularized PLGA TIPS microcarriers were cultured in transwell inserts that simulated the confined *in situ* environment likely to be associated with implanting the product into tissue cavities associated with orthopaedic medicine. The attached cells bridged adjacent microcarriers forming clusters when maintained in differentiation medium. The clustering of cellularized microcarriers did not occur when the samples were maintained under the same physical conditions, indicating the biochemical components in the culture medium provided the pivotal stimuli that led to cell aggregation, differentiation and the formation of the solid 3D constructs. Similar fusion of cellularized microcarriers or micro-objects into larger neo-tissue like constructs has recently been described and investigated for its potential use in other modular bottom-up tissue engineering applications (Chen et al., 2011; Zhou et al., 2016). In their recent work, Zhou et al. reported the formation of macroscopic 3D geometrically shaped cartilage-like composite based on a bottom-up approach using nanofibrous-surface chitosan microcarriers and chondrocytes (Zhou et al., 2016). The cartilage-like composite exhibited phenotypic characteristics of cartilage. In accordance with this, the current study shows the formation of a solid 3D composite tissue construct based on cAdMSC and PLGA microcarriers in both osteogenic and chondrogenic medium, but not in proliferation medium.

The geometrical properties of microspheres offer several benefits when used as cellularized microcarriers intended for *in vivo* delivery, including ease of delivery and provision of evenly distributed interstices between packed microcarriers *in situ* that will allow better diffusion of nutrients compared with less spherical objects. Microspheres in the range of 100–400 μm diameter made from natural or synthetic polymers have been used as cell-microcarriers for a variety of biomedical applications, including cell expansion, biomolecule harvesting and tissue engineering [reviewed in Leong and Wang (2015)]. Cell-laden microcarriers provide a 3D environment *in vitro* that delivers a high surface area: volume ratio and scalability that constitute significant advantages over the traditional 2D monolayer culture (Leong and Wang, 2015). A key feature of the microcarriers investigated in the current study is their potential for use as an implantable temporary cell substrate. Unlike many existing commercially available microcarriers, such as the dextran-based Cytodex3 (Goh et al., 2013) and the polystyrene-based Synthemax III (Hervy et al., 2014), PLGA is biodegradable and can be used to produce products intended for translation into clinical use. PLGA degrades *in vivo* into lactate (salt form of lactic acid) and glycolate (salt form of glycolic acid), which can be completely resorbed by the organism via natural metabolic pathways. The *in vivo* persistence of the microcarriers can be controlled by adjusting parameters that influence PLGA degradation. These include polymer chemistry (e.g., lactide:glycolide ratio, MW, etc.) size and morphology (e.g., shape, surface to volume ratio, porosity, etc.) as well as the environment in which degradation takes place (e.g., pH) [reviewed in Shive and Anderson (1997)]. PLGA already has an excellent clinical safety record and has been successfully used in several medical devices (Athanasiou et al., 1996).

Mesenchymal stromal cells and polymeric microcarriers have been widely used for the expansion of cells when incubated in spinner flasks or other scale up systems. In these circumstances, the emphasis is usually placed on maintaining an MSC phenotype when incubated in proliferation medium during long-term culture (Frauenschuh et al., 2007; Schop et al., 2008). Culturing MSC cell-laden microcarriers in differentiation medium and their implementation as building blocks to form macro-sized constructs for tissue engineering applications has only recently been reported, particularly for bone and cartilage applications (Yang et al., 2007; Chen et al., 2011; Georgi et al., 2014). In accordance with these studies, the current study reports the formation of a solid 3D composite tissue construct based on cAdMSC and biodegradable PLGA microcarriers in both osteogenic and chondrogenic medium. The constructs exhibited phenotypic characteristics indicative of the osteogenic and chondrogenic differentiation, respectively, with ECM production.

Various biomaterial constructs have been developed for mechanically demanding tissue engineering applications. Examples include hydrogels and electrospun fibrous matrices for articular cartilage replacement and bone fillers (Laurencin et al., 1999; Liu et al., 2017). However, the majority of these studies characterize the mechanical properties of the biomaterials before seeding the cells. It is likely that the addition of cells will affect the mechanical properties of the constructs, as shown by studies comparing cell-laden and acellular scaffolds (Silva-Correia et al., 2013; Pangesty et al., 2016). Thus, an increasing number of studies are now focusing on characterizing the mechanical properties of cell-laden biomaterial constructs, including electrospun fibrous mats (Eap et al., 2015; Pangesty et al., 2016), hydrogels (Huang et al., 2008), and microcarrier-based 3D constructs (Zhou et al., 2016). Although more challenging to characterize, such “living” biomaterial constructs resemble more closely the target tissue where cells infiltrate and secrete ECM molecules giving rise to the unique structures reflected by the distinct mechanical characteristics of these tissues.

Rigid disc structures with similar tactile properties were formed for all donor samples following incubation in osteogenic medium that were not observed following incubation in proliferation medium. Viscoelasticity is an important characteristic property of bone, mainly stemming from its distinct hierarchical structure (Lakes et al., 1979). DMA data from the cellularized disc derived from one donor incubated in osteogenic medium for 21 days exhibited viscoelastic characteristics throughout the full range of frequencies investigated in compression mode. Both storage and loss moduli were shown to be frequency dependent. However, the mean values obtained were in the range of MPa whereas the respective value from cortical bone specimens measured with DMA are typically in GPA size range (Abdel-Wahab et al., 2011). Although there are no studies reporting DMA analysis on cancellous trabecular bone, the reported Young’s modulus measured by different techniques is in the range of 50–500 MPa (Bandyopadyay-Ghosh, 2008). The composite construct established in osteogenic medium in the current study had viscoelastic properties much lower than these values, indicating it would not provide adequate mechanical strength as standalone bone replacement in their current format. However, the storage modulus for the TIPS construct (up to 2.5 MPa) was greater than mechanically soft materials, such as injectable hydrogels synthesized from natural-based biomaterials (Geng et al., 2012; Fathi et al., 2014), designed for load-bearing tissue engineering applications. Further studies are required to validate whether similar mechanical properties are achieved with samples generated from multiple cell donors following osteogenic differentiation.

Viscoelasiticity is also an important property of articular cartilage (Sophia Fox et al., 2009). DMA has been used to determine the viscoelastic properties of articular cartilage at a range of frequencies associated with physiological frequencies, ranging from a standard walking pace (1 Hz), to healthy gait heel strike relevant frequencies (3–5 Hz) (Sadeghi et al., 2015) and pathological conditions (Espino et al., 2014; Temple et al., 2016). All studies have reported that the storage modulus is greater than the loss modulus and that the former is frequency-dependent (Fulcher et al., 2009; Espino et al., 2014; Sadeghi et al., 2015). However, the loss modulus is measured as frequency-dependent when off-bone (Temple et al., 2016), but frequency-independent when on-bone (Espino et al., 2014). In our study, cellularized PLGA TIPS microspheres following incubation in chondrogenic medium for 21 days exhibited viscoelastic characteristics throughout the full range of frequencies investigated via DMA in compression mode. The frequency range that was measured included physiological frequencies. Both storage and loss moduli were shown to be frequency-dependent, with the former greater than the latter for all frequencies tested. Our study shows that the disc-like structures formed *in vitro* have similar behavior to the human and bovine cartilage measured with DMA, albeit one order of magnitude smaller (Temple et al., 2016).

There is a strong clinical demand for osteogenic bone grafting and filling scaffolds (Roseti et al., 2017). Current clinical practice involves the use of autograft or allograft bone “putties” which eventually recellularise by infiltration and remodeling from surrounding osteoblasts. However, autografts involve secondary surgery with donor site morbidity, while allografts impose the risk of immune reaction (Haugen et al., 2019). There are now many synthetic alternatives to autografted bone but none have yet been shown to give improved outcomes over autografts (Haugen et al., 2019). The requirements for an ideal bone graft are porosity, biocompatible/osteoconductive surface, controlled biodegradability and mechanical strength (Haugen et al., 2019). A synthetic bone graft that meets all these characteristics is still lacking. The cellularized TIPS microcarrier constructs described in the current study are shown to provide viable, pre-differentiated osteoblast-like cells in a biocompatible matrix that could be used to facilitate bone regeneration. The constructs that form via a modular bottom-up TE approach could potentially be used for various defect sizes and shapes for different orthopaedic applications. Furthermore, the use of donor material from veterinary patients is a valuable tool to help investigate new regenerative medicine technologies toward clinical application in both humans and animals in orthopaedic regenerative medicine (de Bakker et al., 2013). This could be particularly helpful when evaluating the efficacy of new technology in spontaneous, naturally occurring disease, rather than reliance on artificially induced pathology conventionally utilized in non-clinical studies.

In conclusion, the current proof-of-concept study indicates that PLGA porous microcarriers can be used in modular bottom-up tissue engineering approach for the delivery of cellularized constructs intended for orthopaedic applications. This could offer a novel modular bottom-up tissue engineering approach for treating osteotomies and other orthopaedic-related defects requiring the replacement of bone or cartilage tissue.

## Data Availability Statement

The raw data supporting the conclusions of this article will be made available by the authors, without undue reservation.

## Ethics Statement

Canine adipose-derived MSC were isolated from falciform and inguinal adipose tissue from six canine donors with different background breeds. All samples were collected as part of procedures being performed for preparation of cells for unrelated therapeutic clinical applications. Excess cells from the unrelated therapeutic preparations were collected with the informed consent of the owners.

## Author Contributions

CS designed and conducted the experiments involving cAdMSC and TIPS microcarriers, analyzed the data, and drafted the manuscript. MV performed the experiments and analyzed the data involving AdMSC and the TIPS microcarriers. AG established the cAdMSC and performed the differentiation experiments. ZK-E performed the experiments of DMA and SEM. JM provided the cAdMSC and edited the manuscript. RD conceived the study, designed the experiments, interpreted results, and edited the manuscript. All authors reviewed the manuscript.

## Conflict of Interest

AG and JM are employed by company Cell Therapy Sciences Ltd. The remaining authors declare that the research was conducted in the absence of any commercial or financial relationships that could be construed as a potential conflict of interest.

## References

[B1] Abdel-Wahab A. A. Khurshid A. Silberschmidt V. V. (2011). Analysis of anisotropic viscoelastoplastic properties of cortical bone tissues. *J. Mech. Behav. Biomed. Mater.* 4 807–820. 10.1016/j.jmbbm.2010.10.001 21565728

[B2] Ashammakhi N. Ahadian S. Pountos I. Hu S. K. Tellisi N. Bandaru P. (2019). In situ three-dimensional printing for reparative and regenerative therapy. *Biomed. Microdevices* 21 1–6. 10.1007/s10544-019-0372-2 30955134

[B3] Athanasiou K. A. Niederauer G. G. Agrawal C. M. (1996). Sterilization, toxicity, biocompatibility and clinical applications of polylactic acid/polyglycolic acid copolymers. *Biomaterials* 17 93–102. 10.1016/0142-9612(96)85754-1 8624401

[B4] Bandyopadyay-Ghosh S. (2008). Bone as a collagen-hydroxyapatite composite and its repair. *Trends Biomater. Artif. Organs* 22 116–124.

[B5] Blaker J. J. Knowles J. C. Day R. M. (2008). Novel fabrication techniques to produce microspheres by thermally induced phase separation for tissue engineering and drug delivery. *Acta Biomater.* 4 264–272. 10.1016/j.actbio.2007.09.011 18032120

[B6] Bouffi C. Thomas O. Bony C. Giteau A. Venier-Julienne M. C. Jorgensen C. (2010). The role of pharmacologically active microcarriers releasing TGF-β3 in cartilage formation in vivo by mesenchymal stem cells. *Biomaterials* 31 6485–6493. 10.1016/j.biomaterials.2010.05.013 20570347

[B7] Caplan A. I. (1991). Mesenchymal stem cells. *J. Orthop. Res.* 9 641–650. 1870029 10.1002/jor.1100090504

[B8] Caplan A. I. (2007). Adult mesenchymal stem cells for tissue engineering versus regenerative medicine. *J. Cell. Physiol.* 213 341–347. 10.1002/jcp.21200 17620285

[B9] Chen M. Wang X. Ye Z. Zhang Y. Zhou Y. Tan W. S. (2011). A modular approach to the engineering of a centimeter-sized bone tissue construct with human amniotic mesenchymal stem cells-laden microcarriers. *Biomaterials* 32 7532–7542. 10.1016/j.biomaterials.2011.06.054 21774980

[B10] Correia C. R. Nadine S. Mano J. F. (2020). Cell encapsulation systems toward modular tissue regeneration: from immunoisolation to multifunctional devices. *Adv. Funct. Mater.* 30:1908061 10.1002/adfm.201908061

[B11] de Bakker E. Van Ryssen B. De Schauwer C. Meyer E. (2013). Canine mesenchymal stem cells: state of the art, perspectives as therapy for dogs and as a model for man. *Vet. Q.* 33 225–233. 10.1080/01652176.2013.873963 24404887

[B12] Dikina A. D. Alt D. S. Herberg S. McMillan A. Strobel H. A. Zheng Z. (2018). A modular strategy to engineer complex tissues and organs. *Adv. Sci.* 5 1–17. 10.1002/advs.201700402 29876200 PMC5978945

[B13] Dimarino A. M. Caplan A. I. Bonfield T. L. (2013). Mesenchymal stem cells in tissue repair. *Front. Immunol.* 4:201. 10.3389/fimmu.2013.00201 24027567 PMC3761350

[B14] Du Y. Lo E. Ali S. Khademhosseini A. (2008). Directed assembly of cell-laden microgels for fabrication of 3D tissue constructs. *Proc. Natl. Acad. Sci. U.S.A.* 105 9522–9527. 10.1073/pnas.0801866105 18599452 PMC2474514

[B15] Eap S. Keller L. Schiav J. Huck O. Jacomine L. Fioretti F. (2015). A living thick nanofibrous implant bifunctionalized with active growth factor and stem cells for bone regeneration. *Int. J. Nanomedicine* 10 1061–1075. 10.2147/IJN.S72670 25709432 PMC4327569

[B16] Elloumi-Hannachi I. Yamato M. Okano T. (2010). Cell sheet engineering: a unique nanotechnology for scaffold-free tissue reconstruction with clinical applications in regenerative medicine. *J. Intern. Med.* 267 54–70. 10.1111/j.1365-2796.2009.02185.x 20059644

[B17] Espino D. M. Shepherd D. E. Hukins D. W. (2014). Viscoelastic properties of bovine knee joint articular cartilage: dependency on thickness and loading frequency. *BMC Musculoskelet. Disord.* 15:205. 10.1186/1471-2474-15-205 24929249 PMC4068975

[B18] Fathi A. Mithieux S. M. Wei H. Chrzanowski W. Valtchev P. Weiss A. S. (2014). Elastin based cell-laden injectable hydrogels with tunable gelation, mechanical and biodegradation properties. *Biomaterials* 35 5425–5435. 10.1016/j.biomaterials.2014.03.026 24731705 PMC4419780

[B19] Fernandez J. G. Khademhosseini A. (2010). Micro-masonry: construction of 3D structures by microscale self-assembly. *Adv. Mater.* 22 2538–2541. 10.1002/adma.200903893 20440697 PMC2957829

[B20] Fleischer S. Shapira A. Feiner R. Dvir T. (2017). Modular assembly of thick multifunctional cardiac patches. *Proc. Natl. Acad. Sci. U.S.A.* 114 1898–1903. 10.1073/pnas.1615728114 28167795 PMC5338434

[B21] Foong K. S. Patel R. Forbes A. Day R. M. (2010). Anti-tumor necrosis factor-alpha-loaded microspheres as a prospective novel treatment for crohn’s disease fistulae. *Tissue Eng. Part C Methods* 16 855–864. 10.1089/ten.tec.2009.0599 19886803

[B22] Frauenschuh S. Reichmann E. Ibold Y. Goetz P. M. Sittinger M. Ringe J. (2007). A microcarrier-based cultivation system for expansion of primary mesenchymal stem cells. *Biotechnol. Prog.* 23 187–193. 10.1021/bp060155w 17269687

[B23] Fulcher G. R. Hukins D. W. L. Shepherd D. E. T. (2009). Viscoelastic properties of bovine articular cartilage attached to subchondral bone at high frequencies. *BMC Musculoskelet. Disord.* 10:61. 10.1186/1471-2474-10-61 19497105 PMC2698871

[B24] Geng X. Mo X. Fan L. Yin A. Fang J. (2012). Hierarchically designed injectable hydrogel from oxidized dextran, amino gelatin and 4-arm poly(ethylene glycol)-acrylate for tissue engineering application. *J. Mater. Chem.* 22 25130–25139. 10.1039/c2jm34737g

[B25] Georgi N. Van Blitterswijk C. Karperien M. (2014). Mesenchymal stromal/stem cell-or chondrocyte-seeded microcarriers as building blocks for cartilage tissue engineering. *Tissue Eng. Part A* 20 2513–2523. 10.1089/ten.tea.2013.0681 24621188

[B26] Goh T. K.-P. Zhang Z.-Y. Chen A. K.-L. Reuveny S. Choolani M. Chan J. K. Y. (2013). Microcarrier culture for efficient expansion and osteogenic differentiation of human fetal mesenchymal stem cells. *Biores. Open Access* 2 84–97. 10.1089/biores.2013.0001 23593561 PMC3620494

[B27] Gualandi C. (2011). *Porous Polymeric Bioresorbable Scaffolds for Tissue Engineering.* Berlin: Springer.

[B28] Guillotin B. Guillemot F. (2011). Cell patterning technologies for organotypic tissue fabrication. *Trends Biotechnol.* 29 183–190. 10.1016/j.tibtech.2010.12.008 21256609

[B29] Haugen H. J. Lyngstadaas S. P. Rossi F. Perale G. (2019). Bone grafts: which is the ideal biomaterial? *J. Clin. Periodontol.* 46 92–102. 10.1111/jcpe.13058 30623986

[B30] Hervy M. Weber J. L. Pecheul M. Dolley-Sonneville P. Henry D. Zhou Y. (2014). Long term expansion of bone marrow-derived hMSCs on novel synthetic microcarriers in xeno-free, defined conditions. *PLoS One* 9:e92120. 10.1371/journal.pone.0092120 24638103 PMC3956887

[B31] Hong N. Yang G. H. Lee J. H. Kim G. H. (2018). 3D bioprinting and its in vivo applications. *J. Biomed. Mater. Res. Part B Appl. Biomater.* 106 444–459. 10.1002/jbm.b.33826 28106947

[B32] Horwitz E. M. Prockop D. J. Fitzpatrick L. A. Koo W. W. Gordon P. L. Neel M. (1999). Transplantability and therapeutic effects of bone marrow-derived mesenchymal cells in children with osteogenesis imperfecta. *Nat. Med.* 5 309–313. 10086387 10.1038/6529

[B33] Huang A. H. Yeger-McKeever M. Stein A. Mauck R. L. (2008). Tensile properties of engineered cartilage formed from chondrocyte- and MSC-laden hydrogels. *Osteoarthr. Cartil.* 16 1074–1082. 10.1016/j.joca.2008.02.005.TENSILE 18353693 PMC2601559

[B34] Jaklenec A. Hinckfuss A. Bilgen B. Ciombor D. M. Aaron R. Mathiowitz E. (2008). Sequential release of bioactive IGF-I and TGF-β1 from PLGA microsphere-based scaffolds. *Biomaterials* 29 1518–1525. 10.1016/j.biomaterials.2007.12.004 18166223

[B35] Joyce N. Annett G. Wirthlin L. Olson S. Bauer G. Nolta J. (2010). Mesenchymal stem cells for the treatment of neurodegenerative disease. *Regen Med.* 5 933–946. 10.2217/rme.10.72.Mesenchymal 21082892 PMC3017479

[B36] Kassem M. Ankersen L. Eriksen E. F. Clark B. F. Rattan S. I. (1997). Demonstration of cellular aging and senescence in serially passaged long-term cultures of human trabecular osteoblasts. *Osteoporos. Int.* 7 514–524. 10.1007/BF02652556 9604046

[B37] Krešić N. Šimić I. Lojkić I. Bedeković T. (2017). Canine adipose derived mesenchymal stem cells transcriptome composition alterations: a step towards standardizing therapeutic. *Stem Cells Int.* 2017:4176292. 10.1155/2017/4176292 28246532 PMC5299202

[B38] Lakes R. S. Katz L. Sternstein S. S. (1979). Viscoelastic properties of wet cortical bone–I. Torsional and biaxial studies. *J. Biomech.* 12 657–678. 489634 10.1016/0021-9290(79)90016-2

[B39] Laurencin C. T. Ambrosio A. M. Borden M. D. Cooper J. A. (1999). Tissue engineering: orthopedic applications. *Annu. Rev. Biomed. Eng.* 1 19–46. 11701481 10.1146/annurev.bioeng.1.1.19

[B40] Lazarus H. M. Haynesworth S. E. Gerson S. L. Rosenthal N. S. Caplan A. I. (1995). Ex vivo expansion and subsequent infusion of human bone marrow-derived stromal progenitor cells (mesenchymal progenitor cells): implications for therapeutic use. *Bone Marrow Transpl.* 16 557–564. 8528172

[B41] Leferink A. Schipper D. Arts E. Vrij E. Rivron N. Karperien M. (2014). Engineered micro-objects as scaffolding elements in cellular building blocks for bottom-up tissue engineering approaches. *Adv. Mater.* 26 2592–2599. 10.1002/adma.201304539 24395427

[B42] Leferink A. M. Tibbe M. P. Bossink E. G. B. M. de Heus L. E. van Vossen H. van den Berg A. (2019). Shape-defined solid micro-objects from poly(d,l-lactic acid) as cell-supportive counterparts in bottom-up tissue engineering. *Mater. Today Bio* 4:100025. 10.1016/j.mtbio.2019.100025 32159154 PMC7061620

[B43] Leong W. Wang D. A. (2015). Cell-laden polymeric microspheres for biomedical applications. *Trends Biotechnol.* 33 653–666. 10.1016/j.tibtech.2015.09.003 26475118

[B44] Liew A. Brien T. O. (2012). Therapeutic potential for mesenchymal stem cell transplantation in critical limb ischemia. *Stem Cell Res Ther.* 3:28. 10.1186/scrt119 PMC3580466 22846185

[B45] Lim R. Ricardo S. D. Sievert W. (2017). Cell-based therapies for tissue fibrosis. *Front. Pharmacol.* 8:633. 10.3389/fphar.2017.00633 29033833 PMC5626978

[B46] Liu M. Zeng X. Ma C. Yi H. Ali Z. Mou X. (2017). Injectable hydrogels for cartilage and bone tissue engineering. *Bone Res.* 5:17014. 10.1038/boneres.2017.14 28584674 PMC5448314

[B47] LoFurno D. Mannino G. Giuffrida R. (2018). Functional role of mesenchymal stem cells in the treatment of chronic neurodegenerative diseases. *J. Cell Physiol.* 233 3982–3999. 10.1002/jcp.26192 28926091

[B48] McGuigan A. P. Sefton M. V. (2006). Vascularized organoid engineered by modular assembly enables blood perfusion. *Proc. Natl. Acad. Sci. U.S.A.* 103 11461–11466. 10.1073/pnas.0602740103 16864785 PMC1544192

[B49] Morille M. Toupet K. Montero-Menei C. N. Jorgensen C. Noël D. (2016). PLGA-based microcarriers induce mesenchymal stem cell chondrogenesis and stimulate cartilage repair in osteoarthritis. *Biomaterials* 88 60–69. 10.1016/j.biomaterials.2016.02.022 26945456

[B50] Morille M. Van-Thanh T. Garric X. Cayon J. Coudane J. Noël D. (2013). New PLGA-P188-PLGA matrix enhances TGF-β3 release from pharmacologically active microcarriers and promotes chondrogenesis of mesenchymal stem cells. *J. Control. Release* 170 99–110. 10.1016/j.jconrel.2013.04.017 23648834

[B51] Murphy S. V. Atala A. (2014). 3D bioprinting of tissues and organs. *Nat. Biotechnol.* 32 773–785. 10.1038/nbt.2958 25093879

[B52] Norotte C. Marga F. S. Niklason L. E. Forgacs G. (2009). Scaffold-free vascular tissue engineering using bioprinting. *Biomaterials* 30 5910–5917. 10.1016/j.biomaterials.2009.06.034 19664819 PMC2748110

[B53] Pangesty A. I. Arahira T. Todo M. (2016). Characterization of tensile mechanical behavior of MSCs/PLCL hybrid layered sheet. *J. Funct. Biomater.* 7:14. 10.3390/jfb7020014 27271675 PMC4932471

[B54] Penna C. Perrelli M. G. Karam J. P. Angotti C. Muscari C. Montero-Menei C. N. (2013). Pharmacologically active microcarriers influence VEGF-A effects on mesenchymal stem cell survival. *J. Cell. Mol. Med.* 17 192–204. 10.1111/j.1582-4934.2012.01662.x 23305078 PMC3823149

[B55] PromoCellGmbH (2015). *Chondrogenic Differentiation and Analysis of MSC - PromoCell.* Available online at: https://www.promocell.com/f/2017/11/Chondrogenic_Differentiation_and_Analysis_of_MSC-1.pdf (accessed June 17, 2020).

[B56] Qadura M. Terenzi D. C. Verma S. Al-Omran M. Hess D. A. (2018). Concise review: cell therapy for critical limb ischemia: an integrated review of preclinical and clinical studies. *Stem Cells* 36 161–171. 10.1002/stem.2751 29226477

[B57] Roseti L. Parisi V. Petretta M. Cavallo C. Desando G. Bartolotti I. (2017). Scaffolds for Bone Tissue Engineering: State of the art and new perspectives. *Mater. Sci. Eng. C Mater. Biol. Appl.* 78, 1246–1262. 10.1016/j.msec.2017.05.017 28575964

[B58] Sadeghi H. Espino D. M. Shepherd D. E. T. (2015). Variation in viscoelastic properties of bovine articular cartilage below, up to and above healthy gait-relevant loading frequencies. *Proc. Inst. Mech. Eng. Part H* 229 115–123. 10.1177/0954411915570372 25767149 PMC4456430

[B59] Schop D. Janssen F. Borgart E. de Bruijn J. van Dijkhuizen-Radersma R. (2008). Expansion of mesenchymal stem cells using a microcarrier-based cultivation system: growth and metabolism. *J. Tissue Eng. Regen. Med.* 2 126–135. 10.1002/term 18348332

[B60] Shive M. S. Anderson J. M. (1997). Biodegradation and biocompatibility of PLA and PLGA microspheres. *Adv. Drug Deliv. Rev.* 28 5–24. 10.1016/j.addr.2012.09.004 10837562

[B61] Silva-Correia J. Gloria A. Oliveira M. B. Mano J. F. Oliveira J. M. Ambrosio L. (2013). Rheological and mechanical properties of acellular and cell-laden methacrylated gellan gum hydrogels. *J. Biomed. Mater. Res. Part A* 101 3438–3446. 10.1002/jbm.a.34650 23568694

[B62] Simitzi C. Hendow E. Li Z. Day R. M. (2020). Promotion of proangiogenic secretome from mesenchymal stromal cells via hierarchically structured biodegradable microcarriers. *Adv. Biosyst.* 4:e2000062. 10.1002/adbi.202000062 32511898 PMC8425330

[B63] Sittinger M. Hutmacher D. W. Risbud M. V. (2004). Current strategies for cell delivery in cartilage and bone regeneration. *Curr. Opin. Biotechnol.* 15 411–418. 10.1016/j.copbio.2004.08.010 15464370

[B64] Skardal A. Zhang J. Prestwich G. D. (2010). Bioprinting vessel-like constructs using hyaluronan hydrogels crosslinked with tetrahedral polyethylene glycol tetracrylates. *Biomaterials* 31 6173–6181. 10.1016/j.biomaterials.2010.04.045 20546891

[B65] Sophia Fox A. J. Bedi A. Rodeo S. A. (2009). The basic science of articular cartilage: Structure, composition, and function. *Sports Health* 1 461–468. 10.1177/1941738109350438 23015907 PMC3445147

[B66] Temple D. K. Cederlund A. A. Lawless B. M. Aspden R. M. Espino D. M. (2016). Viscoelastic properties of human and bovine articular cartilage: a comparison of frequency-dependent trends. *BMC Musculoskelet. Disord.* 17:419. 10.1186/s12891-016-1279-1 27716169 PMC5054593

[B67] Urciuolo F. Imparato G. Totaro A. Netti P. A. (2013). Building a tissue in vitro from the bottom up: implications in regenerative medicine. *Methodist Debakey Cardiovasc. J.* 9 213–217. 10.14797/mdcj-9-4-213 24298313 PMC3846076

[B68] Wright B. Parmar N. Bozec L. Aguayo S. D. Day R. M. (2015). A simple and robust method for pre-wetting poly (lactic-co-glycolic) acid microspheres. *J Biomater Appl.* 30 147–159. 10.1177/0885328215577297 25791685 PMC4509882

[B69] Yang Y. Rossi F. M. V. Putnins E. E. (2007). Ex vivo expansion of rat bone marrow mesenchymal stromal cells on microcarrier beads in spin culture. *Biomaterials* 28 3110–3120. 10.1016/j.biomaterials.2007.03.015 17433434

[B70] Yong K. W. Choi J. R. Mohammadi M. Mitha A. P. Sanati-Nezhad A. Sen A. (2018). Mesenchymal stem cell therapy for ischemic tissues. *Stem Cells Int.* 2018:8179075. 10.1155/2018/8179075 PMC6196793 30402112

[B71] Zhang L. Su P. Xu C. Yang J. Yu W. Huang D. (2010). Chondrogenic differentiation of human mesenchymal stem cells: A comparison between micromass and pellet culture systems. *Biotechnol. Lett.* 32 1339–1346. 10.1007/s10529-010-0293-x 20464452

[B72] Zhou Y. Gao H. L. Shen L. L. Pan Z. Mao L. B. Wu T. (2016). Chitosan microspheres with an extracellular matrix-mimicking nanofibrous structure as cell-carrier building blocks for bottom-up cartilage tissue engineering. *Nanoscale* 8 309–317. 10.1039/c5nr06876b 26610691

