# Supplementary material for: Modular Orthopaedic Tissue Engineering With Implantable Microcarriers and Canine Adipose-Derived Mesenchymal Stromal Cells
Source: Front Bioeng Biotechnol. 2020 Jul 22;8:816. doi: 10.3389/fbioe.2020.00816 (PMC7388765; doi:10.3389/fbioe.2020.00816)
Supplement: Supplementary file 1 [file Image_1.pdf]

Supplementary Material

1.1 Supplementary Figures

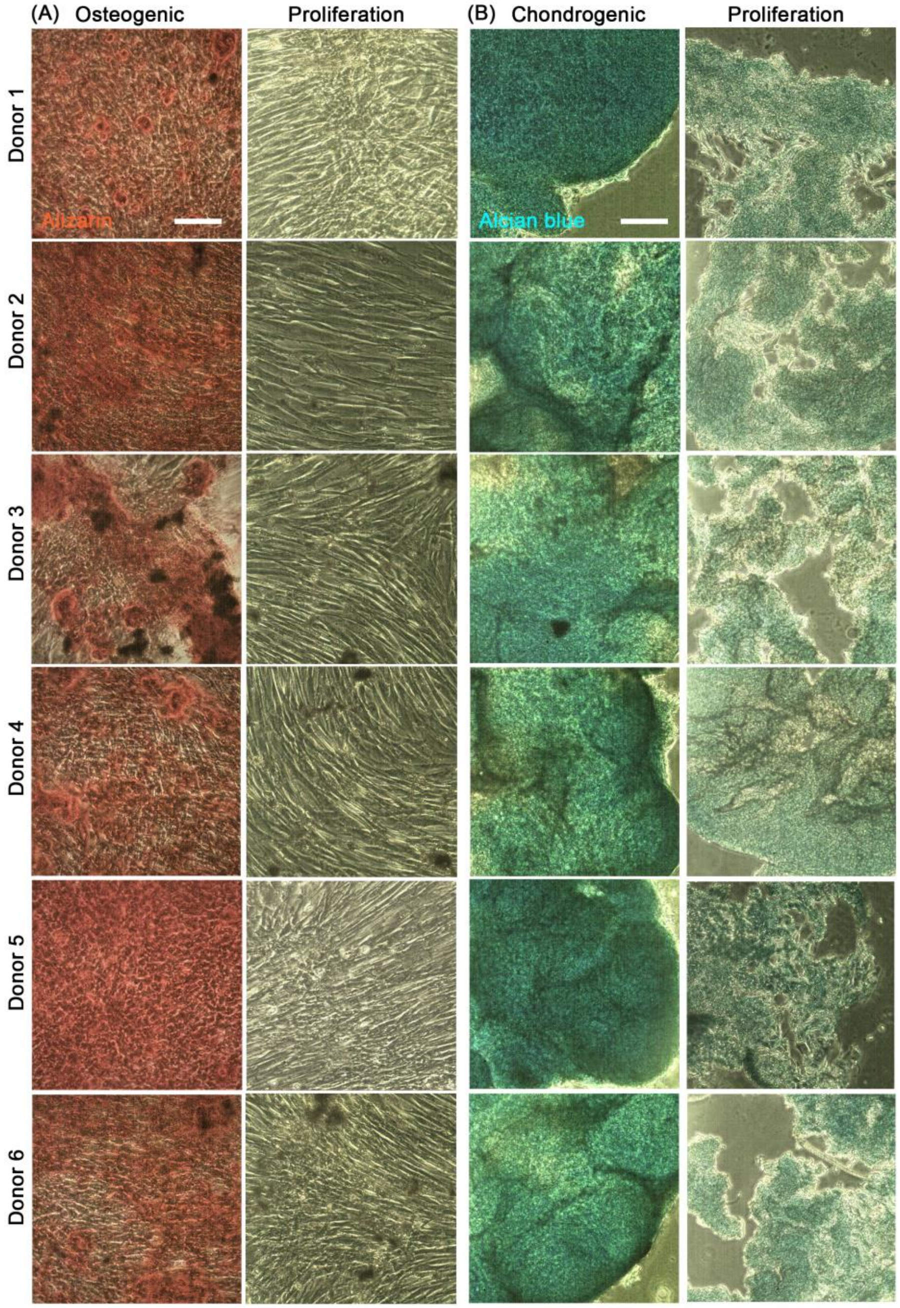

Supplementary Figure 1. (A) Light microscopy of cAdMSC from six donors incubated for 14 days in osteogenic or proliferation medium and stained for Alizarin red (scale bar 100 μm). (B) Light microscopy images of cAdMSC from six donors incubated for 21 days in chondrogenic differentiation or proliferation medium and stained for alcian blue (blue) (scale bar 100 μm).

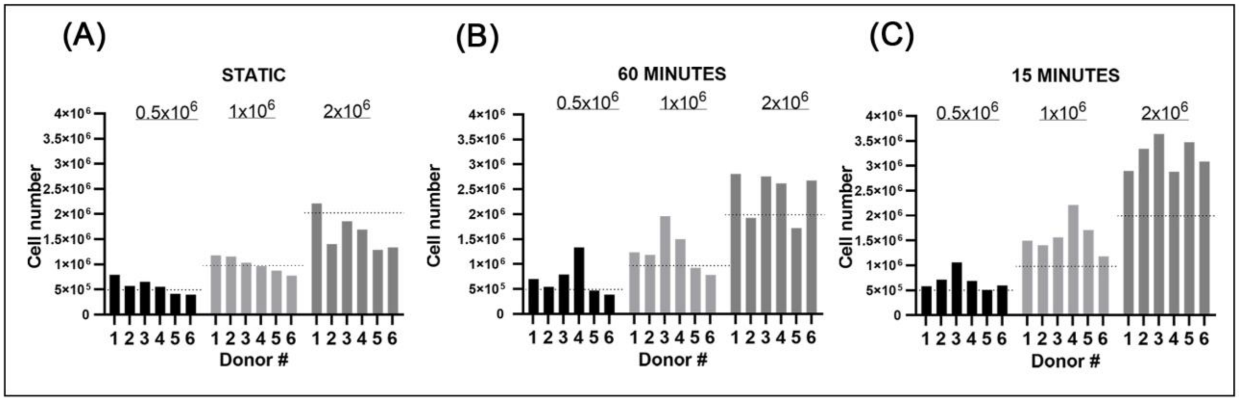

Supplementary Figure 2. Quantification of cAdMSC attachment to the surface of PLGA TIPS microcarriers under different experimental conditions. Cells from six donors were cultured with 50 mg PLGA TIPS microcarriers for 18 hours with either (A) static, (B) intermittent plate shaking for 30 seconds every hour, or (C) intermittent plate shaking for 30 seconds every 15 minutes.
